# Cancerag: structure-aware machine learning pipeline for identifying biased agonists for G-protein coupled receptors

**DOI:** 10.1093/bib/bbaf631.066

**Published:** 2025-12-12

**Authors:** Halleluyah Darasimi Oludele, Pius Kwasi Sam, Joy Olu-Benson, Olaitan Awe

**Affiliations:** University of Ibadan; University of Cape Coast; University of Ibadan; African Society for Bioinformatics and Computational Biology

## Abstract

**Background:**

biased agonism in GPCRs enables selective G protein versus β-arrestin pathway activation, offering therapeutic advantages. GPER modulates oncogenic MAPK and PI3K/Akt signaling in cancer^1^. However, predicting signaling bias remains challenging.

**Methods:**

we developed a structure-aware machine learning pipeline integrating chemical and receptor features to predict GPCR bias. Biased agonists from BiasDB^2^ across 61 GPCRs were supplemented with unbiased ligands from ChEMBL and BindingDB. After Lipinski filtering and PAINS removal, RDKit extracted 200+ molecular descriptors including TPSA, logP, and ECFP4. Automated docking with GPCRdb^3^ structures and AlphaFold2 models generated interaction metrics. K-Means, DBSCAN, Random Forest, and Gradient Boosting with SMOTE balancing and Boruta feature selection were implemented. SHAP^4^ analysis quantified feature contributions.

**Results:**

anticipated >80% cross-validation accuracy with balanced performance. SHAP^4^ reveals scaffold and interaction motifs associated with pathway selectivity. Validation on GPER ligands demonstrates predictive capability. The pipeline accepts receptor-ligand pairs and outputs predicted bias with feature attribution, enabling batch screening.

**Conclusion:**

this structure-aware approach integrates ligand chemistry, receptor structure, and explainable AI^4^ to advance GPCR bias prediction, accelerating rational drug discovery.

**Acknowledgement:**

African Society for Bioinformatics and Computational Biology for the Omics Codeathon.

**References:**

1. Barton M, Prossnitz ER. ‘GPER in health and disease.’ Nature Reviews Endocrinology 2023;19:822–843.

2. Sanchez JE, Sirimulla S. ‘BiasNet: predicting ligand bias toward GPCR signaling.’ Journal of Chemical Information and Modeling 2021;61:4190–4199.

3. Kooistra AJ, Gloriam DE. ‘GPCRdb in 2021: integrating GPCR sequence, structure and function.’ Nucleic Acids Research 2021;49:D335–D343.

4. Lundberg SM, Lee SI. ‘Explainable AI for trees.’ Nature Machine Intelligence 2020;2:56–67.

